# Endoplasmic Reticulum stress-dependent expression of ERO1L promotes aerobic glycolysis in Pancreatic Cancer

**DOI:** 10.7150/thno.45124

**Published:** 2020-07-09

**Authors:** Junfeng Zhang, Jianyu Yang, Chaoyi Lin, Wei Liu, Yanmiao Huo, Minwei Yang, Shu-Heng Jiang, Yongwei Sun, Rong Hua

**Affiliations:** 1 Department of Biliary-Pancreatic Surgery, Ren Ji Hospital, School of Medicine, Shanghai Jiao Tong University, Shanghai 200217, P.R. China.; 2 State Key Laboratory of Oncogenes and Related Genes, Shanghai Cancer Institute, Ren Ji Hospital, School of Medicine, Shanghai Jiao Tong University, Shanghai 200240, P.R. China.

**Keywords:** ERO1α, ERO1A, Aerobic glycolysis, Pancreatic cancer, HIF1α

## Abstract

**Rationale:** Endoplasmic reticulum oxidoreductase 1 alpha (ERO1L) is an endoplasmic reticulum (ER) luminal glycoprotein that has a role in the formation of disulfide bonds of secreted proteins and membrane proteins. Emerging data identify ERO1L as a tumor promoter in a wide spectrum of human malignancies. However, its molecular basis of oncogenic activities remains largely unknown.

**Methods:** Pan-cancer analysis was performed to determine the expression profile and prognostic value of ERO1L in human cancers. The mechanism by which ERO1L promotes tumor growth and glycolysis in pancreatic ductal adenocarcinoma (PDAC) was investigated by cell biological, molecular, and biochemical approaches.

**Results:** ERO1L was highly expressed in PDAC and its precursor pancreatic intraepithelial neoplasia and acts as an independent prognostic factor for patient survival. Hypoxia and ER stress contributed to the overexpression pattern of ERO1L in PDAC. ERO1L knockdown or pharmacological inhibition with EN460 suppressed PDAC cell proliferation *in vitro* and slowed tumor growth *in vivo*. Ectopic expression of wild type ERO1L but not its inactive mutant form EROL-C394A promoted tumor growth. Bioinformatics analyses and functional analyses confirmed a regulatory role of ERO1L on the Warburg effect. Notably, inhibition of tumor glycolysis partially abrogated the growth-promoting activity of ERO1L. Mechanistically, ERO1L-mediated ROS generation was essential for its oncogenic activities. In clinical samples, ERO1L expression was correlated with the maximum standard uptake value (SUVmax) in PDAC patients who received ^18^F-FDG PET/CT imaging preoperatively. Analysis of TCGA cohort revealed a specific glycolysis gene expression signature that is highly correlated with unfolded protein response-related gene signature.

**Conclusion:** Our findings uncover a key function for ERO1L in Warburg metabolism and indicate that targeting this pathway may offer alternative therapeutic strategies for PDAC.

## Introduction

Pancreatic ductal adenocarcinoma (PDAC) is one of the most frequent and deadliest solid tumors. Despite considerable progress made in diagnosis and therapy, the clinical outcome of PDAC is still poor with a 5-year survival rate less than 6% 1. The high mortality in PDAC patients is mainly ascribed to late diagnosis, aggressive local invasion, early metastasis, and a poor sensitivity to chemotherapeutics 2, 3. Therefore, elucidating the underlying molecular mechanisms and biomarkers for PDAC progression is urgently needed to facilitate early diagnosis and treatment of this deadly disease 4.

Endoplasmic reticulum (ER) is an organelle that enables lipid synthesis, calcium storage, and appropriate processing of membrane and secreted proteins for maturation 5. Stress such as low oxygen (hypoxia) or restriction of key nutrients can disrupt protein maturation and cause the accumulation of misfolded proteins in the ER lumen, which triggers unfolded protein response (UPR). Activation of UPR is a mechanism by which tumor cells evade ER stress and promote uncontrolled proliferation 6. Three major signaling pathways (PERK, IRE1α, and ATF6) are activated to increase protein-folding capacity and reduce protein load in the ER. These transcriptional and translational outputs tend to balance ER homeostasis and promote cell survival 7.

Endoplasmic reticulum oxidoreductase 1 alpha (ERO1L) is a flavin adenine nucleotide-containing enzyme that exists in the ER and favors disulfide bond formation of secreted proteins and cell surface proteins by accepting electrons from reduced protein disulfide isomerase (PDI) and passing them on to molecular oxygen 8-11. Emerging evidence has demonstrated that ERO1L is highly expressed in various types of human cancers, and that high expression is associated with worse clinical outcome 12-15. ERO1L overexpression is considered to play a crucial role in tumor malignant phenotypes including tumor growth, metastasis, angiogenesis, and immune escape 16-19. For example, ERO1L inhibits the T cell response by recruiting myeloid-derived suppressor cells via regulation of granulocyte-colony stimulating factor and CXCL1/2 17. ERO1L promotes tumor growth and angiogenesis via oxidative protein folding of vascular endothelial growth factor (VEGF) and enhancement of VEGF mRNA expression 20. Moreover, other reports have also indicated that ERO1L modulates tumor progression by upregulating integrin-β1 expression, MHC Class I molecule expression 21, and activation of the Wnt/β-catenin pathway 16. Therefore, ERO1L is a promising target for cancer therapy. However, the molecular mechanism of ERO1L function in tumor biology remains unclear and warrants further investigation.

In this study, we first deciphered the perspective of ERO1L gene expression profile and prognostic value in human cancers, especially PDAC. By loss- and gain-of-function studies, we revealed that ERO1L favors cell proliferation *in vitro* and facilitates tumor growth *in vivo*. Bioinformatics analyses revealed that ERO1L expression is correlated with Warburg metabolism. Further experiments identified Warburg effect as a new mechanism that plays a role in mediating the growth-promoting effect of ERO1L on PDAC cells.

## Materials and Methods

### Data mining using Oncomine, TCGA, and GTEx

*ERO1L* gene expression was analyzed using microarray gene expression datasets deposited in Oncomine database (https://www.oncomine.org). A combined filter was applied to display the corresponding datasets. The Cancer Type was defined as Pancreatic Cancer and Data Type was mRNA, and Analysis Type was Cancer versus Normal Analysis. The Gene Expression Profiling Interactive Analysis 2 (GEPIA2) database (http://gepia.cancer-pku.cn/index.html) 22, which provides an overview of the TCGA/GTEx data, was used to analyze the expression pattern and prognostic value of *ERO1L* gene across 33 types of human cancers. Abbreviations for several introduced tumor types were shown as follows: ACC, Adrenocortical carcinoma; BLCA, Bladder urothelial carcinoma; CESC, Cervical squamous cell carcinoma and endocervical adenocarcinoma; COAD, Colon adenocarcinoma; ESCA, Esophageal carcinoma; KICH, Kidney chromophobe; KIRC, Kidney renal clear cell carcinoma; KIRP, Kidney renal papillary cell carcinoma; LIHC, Liver hepatocellular carcinoma; LUAD, Lung adenocarcinoma; LUSC, Lung squamous cell carcinoma; MESO, Mesothelioma; PAAD, Pancreatic adenocarcinoma; READ, Rectum adenocarcinoma; STAD, Stomach adenocarcinoma; and THYM, Thymoma. For patients' survival analysis, the log-rank test was used and the result was shown as hazard ratio (HR) with 95% confidence intervals.

### Cell culture and reagents

Human pancreatic cancer cell lines AsPC-1, BxPC-3, Capan-2, CFPAC-1, MiaPaCa-2, PANC-1, Patu8988, and the nonmalignant HPDE cells were all preserved in Shanghai Cancer Institute, Ren Ji Hospital, School of Medicine, Shanghai Jiao Tong University. Cells were cultured in suggested medium according to American Type Culture Collection (ATCC) protocols, supplemented with 10% (v/v) fetal bovine serum (FBS) and 1% (v/v) streptomycin-penicillin (Sigma-Aldrich, Shanghai, China) at 37 °C in a humidified incubator under 5% CO_2_ condition. For hypoxia experiments, cells were subjected to low-oxygen culture (< 1% O_2_) in a hypoxia chamber for 24 h. Galactose (G5388), mannose (M6020), 2-fluoro-deoxy-D-glucose (2-FDG, F5006), and the antioxidant N-acetylcysteine (NAC, A7250) were obtained from Sigma-Aldrich (Shanghai, China). GSK2656157 (S7033), Buthionine-[S, R]-sulfoximine (BSO, S2433), and 2-deoxy-D-glucose (2-DG, S4701) were purchased from Selleck (Shanghai, China). Tunicamycin (Tm, A611129), Thapsigargin (Tg, A616759), and DTT (A620058) were all purchased from Sangon Biotech (Shanghai, China).

### Clinical specimens

A tissue microarray containing 205 PDAC specimens and corresponding non-cancerous tissues were generated as reported previously 23. None of the patients had received radiotherapy, chemotherapy, hormone therapy or other related anti-tumor therapies before surgery. All the patients were provided with written informed consent before enrollment, and the study was approved by the Research Ethics Committee of Ren Ji Hospital, School of Medicine, Shanghai Jiao Tong University.

### HE staining and immunohistochemistry

Hematoxylin and eosin (H&E) staining was performed routinely. Immunohistochemical (IHC) analysis was done as described previously 24. In brief, antigen retrieval was performed after de-paraffinization by boiling at 100 °C for 15 min in 10 mM citrate buffer (pH 6.0). The primary antibodies used in this study were: ERO1L (1:100, Abcam, ab177156), XBP1s (1:200, Cell Signaling Technology, #40435), phospho-eIF2α (1:100, Cell Signaling Technology, #3398), and CHOP (1:200, Abcam, ab179823). The immunoreactivity was visualized with 3,3'-diaminobenzidine tetrahydrochloride (DAB) and counterstained was done with hematoxylin. IHC score was calculated based on the percentage of positive-staining cells: 0-5% scored 0, 6-35% scored 1, 36-70% scored 2, and more than 70% scored 3; and staining intensity: no staining scored 0, weakly staining scored 1, moderately staining scored 2 and strongly staining scored 3. The final score was calculated using the percentage score multiply staining intensity score as follows: “-” for a score of 0-1, “+” for a score of 2-3,“++” for a score of 4-6 and “+++” for a score of > 6. Low expression was defined as a total score < 4 and high expression with a total score ≥ 4. These scores were determined independently by two senior pathologists who were blinded to clinicopathologic data.

### Lentiviral transfection and siRNA-mediated knockdown

Short hairpin RNA (shRNA) against *ERO1L* gene or control vectors were transfected along with a three plasmid system (pPACKH1-GAG, pPACKH1-REV, and pVSV-G) into HEK293T cells using Lipofectamine 2000 (Invitrogen, Carlsbad, CA, USA) according to the manufacturer's instructions. Conditioned medium containing viral particles was harvested at 48 h and 72 h after transfection, and filtered through 0.45-μm filters. Cells were then infected with recombinant lentivirus in the presence of 6 μg/ml polybrene (Sigma-Aldrich, H9268, Shanghai, China). After infection for 48 h, cells were selected with 2 μg/ml puromycin (Gibco, A1113802, USA) for 7 days to eliminate the uninfected cells and thus yield mass populations of puromycin-resistant cells expressing the shRNAs. Full-length *ERO1L* and its inactive mutant C394A were cloned into pCDH-CMV-MCS-EF1-Puro vector to generate pCDH-ERO1L and pCDH-ERO1L-C394A overexpression plasmids, respectively. The specific siRNAs for HIF1α were designed and synthesized in Genepharma (Shanghai, China); the sequence information was shown as follows: si-HIF1α-1, GAGGAAGAACUAAAUCCAAdTdT; si-HIF1α-2, UGAUACCAACAGUAACCAAdTdT; the si-Ctrl was non-homologous to any human genome sequences. Lipofectamine® RNAiMAX reagent (ThermoFisher Scientific, #13778030, USA) was used to conduct siRNA transfection according to the manufacturer's protocols.

### Real-time quantitative PCR

Total RNA was isolated from pancreatic cancer cells using RNAiso Plus (Takara, Japan) and reversely transcribed through PrimeScript RT-PCR kit (Takara, Japan) according to the manufacturer's instructions. Quantitative real-time PCR was performed with SYBR Premix Ex Taq (Takara, Japan) on a 7500 Real-time PCR system (Applied Biosystems, Inc. USA) at the recommended thermal cycling settings: one initial cycle at 95 °C for 2 min followed by 40 cycles of 5 sec at 95 °C and 31 sec at 60 °C. Gene expression was normalized to human *ACTB* gene transcripts. Relative mRNA expression was calculated by the 2^-ΔΔCt^ method. Primer sequences used in this study are shown as follows: *ERO1L* forward, 5′-GGCTGGGGATTCTTGTTTGG-3′; *ERO1L* reverse 5′-AGTAACCACTAACCTGGCAGA-3′; β-actin forward, 5′-ACTCGTCATACTCCTGCT-3′, β-actin reverse, 5′-GAAACTACCTTCAACTCC-3′.

### Western blot analysis

Whole-cell extracts were extracted using a total protein extraction buffer (Beyotime, Shanghai, China) supplemented with protease inhibitors and the protein concentration was measured using a BCA Protein Assay Kit (Pierce Biotechnology, USA). Protein lysates were separated by 6-12% SDS-PAGE and transferred to a nitrocellulose membrane (Millipore, MA, USA). After blocking with 5% skimmed milk, the membrane was probed with one of the following primary antibodies: ERO1L (1:1000, Abcam, ab177156), HIF1α (1: 1000, Abcam, ab113642), BiP (1: 1000, Cell Signaling Technology, #3177), CHOP (1: 1000, Cell Signaling Technology, #2895), phospho-eIF2α (Ser51) (1:1000, Cell Signaling Technology, #3398), eIF2α (1:1000, Cell Signaling Technology, #5324), and β-actin (1:2000, Abcam, ab8226). The next day, the membranes were incubated with species-specific secondary antibodies (ThermoFisher Scientific, USA). Finally, bound secondary antibodies were detected by Odyssey imaging system (LI-COR Biosciences, Lincoln, NE, USA).

### Measurement of extracellular acidification rate (ECAR) and oxygen consumption rate (OCR)

The Seahorse Bioscience XF96 Extracellular Flux Analyzer (Seahorse Bioscience, USA) was used to monitor mitochondrial function and glycolytic capacity with Seahorse XF Cell Mito Stress Test Kit or Glycolysis Stress Test Kit (Seahorse Bioscience, Billerica, MA, USA). In brief, cells were seeded in a XF96-well plate at a density of 4 × 10^4^ per well and allowed to attach overnight. For ECAR assessment, cells were incubated with unbuffered medium followed by a sequential injection of 10 mM glucose, 1 μM oligomycin, and 80 mM 2-DG. The mitochondrial respiration (OCR) was measured by sequential injection of 1 μM oligomycin, carbonyl cyanide 4-(trifluoromethoxy) phenylhydrazone (FCCP) and 2 μM antimycin A and rotenone. The ECAR and OCR value were normalized to the total protein content between groups prior to making comparisons.

### Glucose uptake and lactate production

Briefly, cells were seeded into 6-well culture plates at a density of 3 × 10^5^ per well. The supernatants of the cultured cells were collected after 24 h. The glucose concentration was detected using a glucose assay kit (Sigma-Aldrich, MAK263, USA). Glucose uptake was calculated by deducting the measured glucose level in the culture media from the original glucose concentration (25 mM). Similarly, lactate level was determined using a Lactate Assay Kit (BioVision, K607-100, USA) according to the manufacturer's protocol. Finally, glucose uptake and lactate production were normalized to total protein content which was measured by the Pierce BCA Protein assay (Pierce Biotechnology, USA).

### Measurement of ROS levels

PDAC cells were grown in black 96-well plates overnight at 37 °C. The next day, cells were treated with Tm to induce ER stress. Then the cells were washed with 1 × PBS for three times and DCF-DA (10 mmol/L) in phenol red-free medium was added to the cells and allowed to incubation for 30 min. Finally, the fluorescence was detected using a BioTek fluorescence plate reader.

### Cell viability assay

Cell viability was measured using a Cell Counting Kit-8 (CCK-8, Dojindo Molecular Technologies, Japan). Briefly, cells were seeded into 96-well plates at 3,000 cells per well and incubated overnight. At the indicated time, cells were cultured for 1 h with 10% (v/v) CCK-8 reagent. Then, absorbance at 450 nm was measured by an enzyme-linked immunosorbent assay reader (Bio-Rad Laboratories, Hercules, CA).

### Colony formation assay

Briefly, cells were seeded onto a 6-well plate at a density of 1000 cells per well. The culture medium was replaced every three days. After 12-14 days, the cells were fixed with 4% paraformaldehyde and then stained with 0.1% crystal violet. The numbers of colonies were counted.

### Animal experiments

Balb/c nude mice ages 6 weeks were used for subcutaneous xenograft experiment. Mice were manipulated and housed according to according to the criteria outlined in the “Guide for the Care and Use of Laboratory Animals” prepared by the National Academy of Sciences and published by the National Institutes of Health. In brief, a total of 2 × 10^6^ indicated PDAC cells in 200 ul DMEM medium were injected subcutaneously in the lower back. Four weeks later, mice were sacrificed, tumor tissues were isolated and tumor weight was measured. This study was approved by the Research Ethics Committee of Shanghai Jiao Tong University.

### Statistical analysis

Results were presented as mean ± SD. Continuous variables were compared using two-tailed Student's t-test or one-way ANOVA test. The SPSS 13.0 statistical package software (SPSS, Chicago, IL, USA) or GraphPad Prism (GraphPad Software Inc., San Diego, CA) was used for statistical analyses. Prognostic analysis was conducted by the Kaplan-Meier method and analyzed by the log-rank test. Correlation analysis among genes was determined by Spearman's correlation. A P value less than 0.05 were considered statistically significant.

## Results

### A pan-cancer perspective of *ERO1L* gene expression and prognostic potential

**Figure 1:**
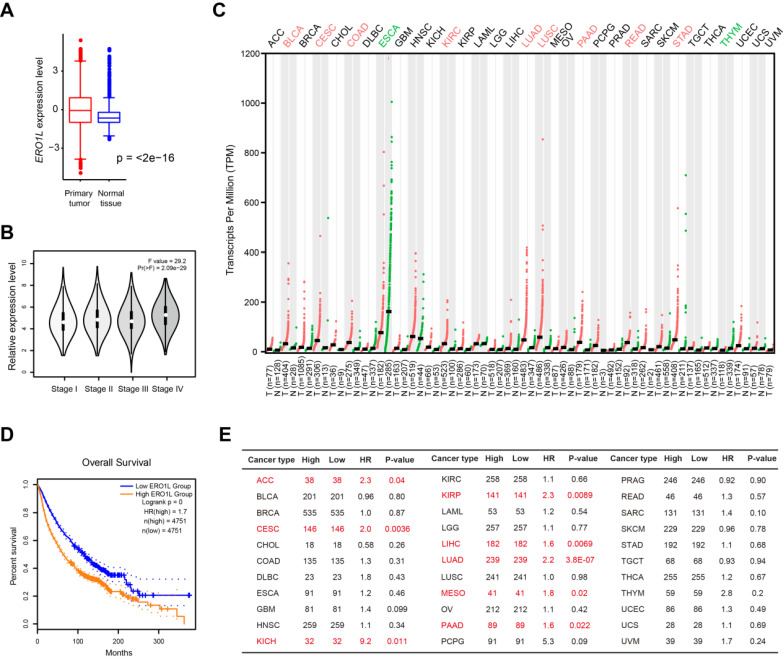
** Pan-cancer perspective of *ERO1L* gene expression profile and prognostic potential.** (**A**) Comparison of *ERO1L* expression level in 9664 tumor tissue samples and 5539 normal cases. Data were derived from TCGA cohort and GTEx. (**B**) Comparison of* ERO1L* expression level in tumor tissue samples with different TNM stage. (**C**) The expression level of ERO1L in different tumor tissues and their normal counterparts. (**D**) Kaplan-Meier graphs showing significant association of ERO1L expression with patients' survival. (**E**) Prognostic analysis of ERO1L in different human cancers. The median expression value of ERO1L was used as a cutoff. HR: hazard ratio. ACC, Adrenocortical carcinoma; BLCA, Bladder urothelial carcinoma; BRCA, Breast invasive carcinoma; CESC, Cervical squamous cell carcinoma and endocervical adenocarcinoma; CHOL, Cholangio carcinoma; COAD, Colon adenocarcinoma; DLBC, Lymphoid neoplasm diffuse large B-cell lymphoma; ESCA, Esophageal carcinoma; GBM, Glioblastoma multiforme; HNSC, Head and neck squamous cell carcinoma; KICH, Kidney chromophobe; KIRC, Kidney renal clear cell carcinoma; KIRP, Kidney renal papillary cell carcinoma; LAML, Acute myeloid leukemia; LGG, Brain lower grade glioma; LIHC, Liver hepatocellular carcinoma; LUAD, Lung adenocarcinoma; LUSC, Lung squamous cell carcinoma; MESO, Mesothelioma; OV, Ovarian serous cystadenocarcinoma; PAAD, Pancreatic adenocarcinoma; PCPG, Pheochromocytoma and paraganglioma; PRAD, Prostate adenocarcinoma; READ, Rectum adenocarcinoma; SARC, Sarcoma; SKCM, Skin cutaneous melanoma; STAD, Stomach adenocarcinoma; TGCT, Testicular germ cell tumors; THCA, Thyroid carcinoma; THYM, Thymoma; UCEC, Uterine corpus endometrial carcinoma; UCS, Uterine carcinosarcoma; UVM, Uveal melanoma.

To define the expression pattern of *ERO1L* gene in human cancers, we used transcriptomic profiles from The Cancer Genome Atlas (TCGA) and the Genotype-Tissue Expression (GTEx) portal. This approach integrates 9664 tumor tissue samples and 5539 normal cases available for expression analysis. Indeed, *ERO1L* mRNA level was significantly elevated in tumor samples compared with normal controls (Figure 1A) and increased from stage I to stage IV in a stepwise manner (Figure 1B). Aside from ESCA and THYM, *ERO1L* expression was dramatically higher in many cancer types compared to matched tissue controls, including BLCA, CSEC, COAD, KIRC, LUAD, LUSC, PAAD, READ, and STAD (Figure 1C). Kaplan-Meier survival analysis showed that *ERO1L* expression was associated with a survival disadvantage with a hazard ratio (HR) of 1.7 (P < 0.0001) across 33 cancer types (Figure 1D). Specifically, *ERO1L* predicted a poor prognosis in ACC (HR = 2.3; P = 0.04), CESC (HR = 2.0; P = 0.0038), KICH (HR = 9.2; P = 0.011), KIRP (HR = 2.3; P = 0.0089), LIHC (HR = 1.6; P = 0.0069), LUAD (HR = 2.2; P < 0.0001), MESO (HR = 1.8; P = 0.02), and PAAD (HR = 1.6; P = 0.022) (Figure 1E). Therefore, *ERO1L* typically featured a significant expression difference between groups and was among the overall survival predictors observed within CESC, LUAD, and PAAD.

### ERO1L is overexpressed in PDAC and predicts a poor prognosis

**Figure 2:**
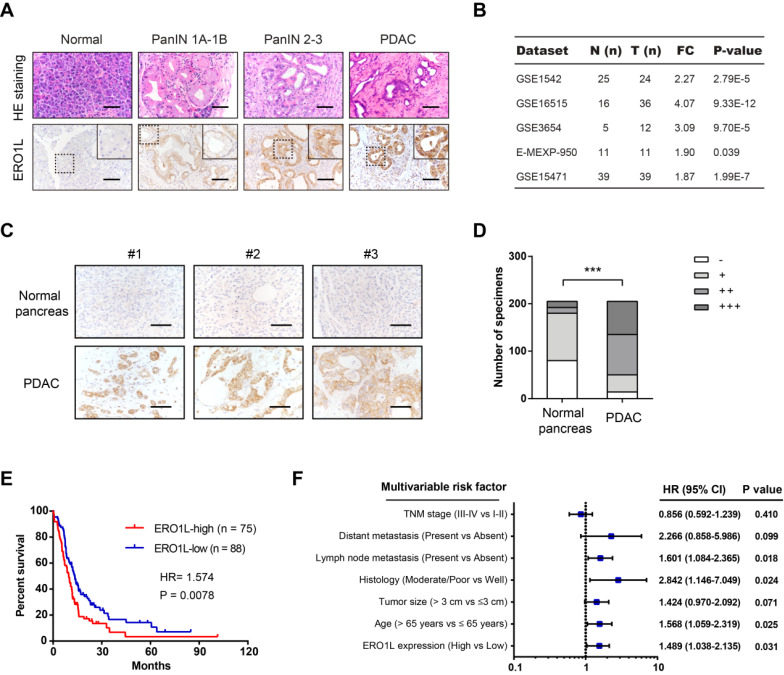
** ERO1L is overexpressed in PDAC and predicts a poor prognosis.** (**A**) Immunohistochemical detection of ERO1L protein expression in normal pancreas from control mice, precursor lesions from Pdx1-Cre; LSL-Kras^G12D/+^ (KC) mice, and tumor lesions from Pdx1-Cre; LSL-Kras^G12D/+^; LSL-Trp53^R172H/+^ (KPC) mice. HE staining was performed to show the representative images of different stages of PanIN lesions; Scale bar: 50 µm. (**B**) The expression pattern of ERO1L in five independent PDAC cohorts. Data were derived from the GEO database. (**C**) Representative images of ERO1L protein expression in a tissue microarray containing 205 pathologist-certified and clinically annotated PDAC specimens from Ren Ji cohort; Scale bar: 50 µm. (**D**) The number of tissue specimens displaying high or low ERO1L staining in paracancerous pancreas and PDAC lesions (Fisher's exact test, **P < 0.01). (**E**) Kaplan-Meier analysis of the overall survival of PDAC patients based on ERO1L protein expression in Ren Ji cohort (log-rank test, P = 0.0078). (**F**) Multivariate Cox regression analyses were performed to identify independent prognostic factors for PDAC survival. All the bars correspond to 95% confidence intervals.

In this study, we selected PAAD from those tumor types for detailed analysis. Pdx1-Cre; LSL-Kras^G12D/+^; LSL-Trp53^R172H/+^ (KPC) mice, characterized by highly accelerated development of pancreatic intraepithelial neoplasia (PanINs) and well differentiated PDAC, are a well-documented preclinical mouse model to study the implications of disease modifiers. Interestingly, we revealed that ERO1L protein expression was elevated in human PanIN and PDAC tissues compared with normal pancreas tissues (Figure 2A). Data mining the GEO database showed that ERO1L is commonly upregulated in PDAC as demonstrated by five independent cohorts (Figure 2B). To further determine the expression pattern of ERO1L in PDAC, we performed immunohistochemical analysis in a large-scale tissue microarray containing 205 pathologist-certified and clinically annotated PDAC samples. As a result, ERO1L was highly and frequently expressed in PDAC tissues compared with paracancerous pancreas tissues (Figure 2C-D). Kaplan-Meier curve showed that the overall survival rate was shorter in PDAC patients with higher ERO1L expression than in those with lower ERO1L expression (Figure 2E). Importantly, ERO1L expression was closely correlated with tumor size (P = 0.002) and histological differentiation (P = 0.033, Table S1). Moreover, univariate and multivariate Cox regression analyses identified ERO1L expression as an independent predictor of overall survival in PDAC patients (Figure 2F and Table S2).

### ERO1L is induced by ER stress in pancreatic cancer

**Figure 3:**
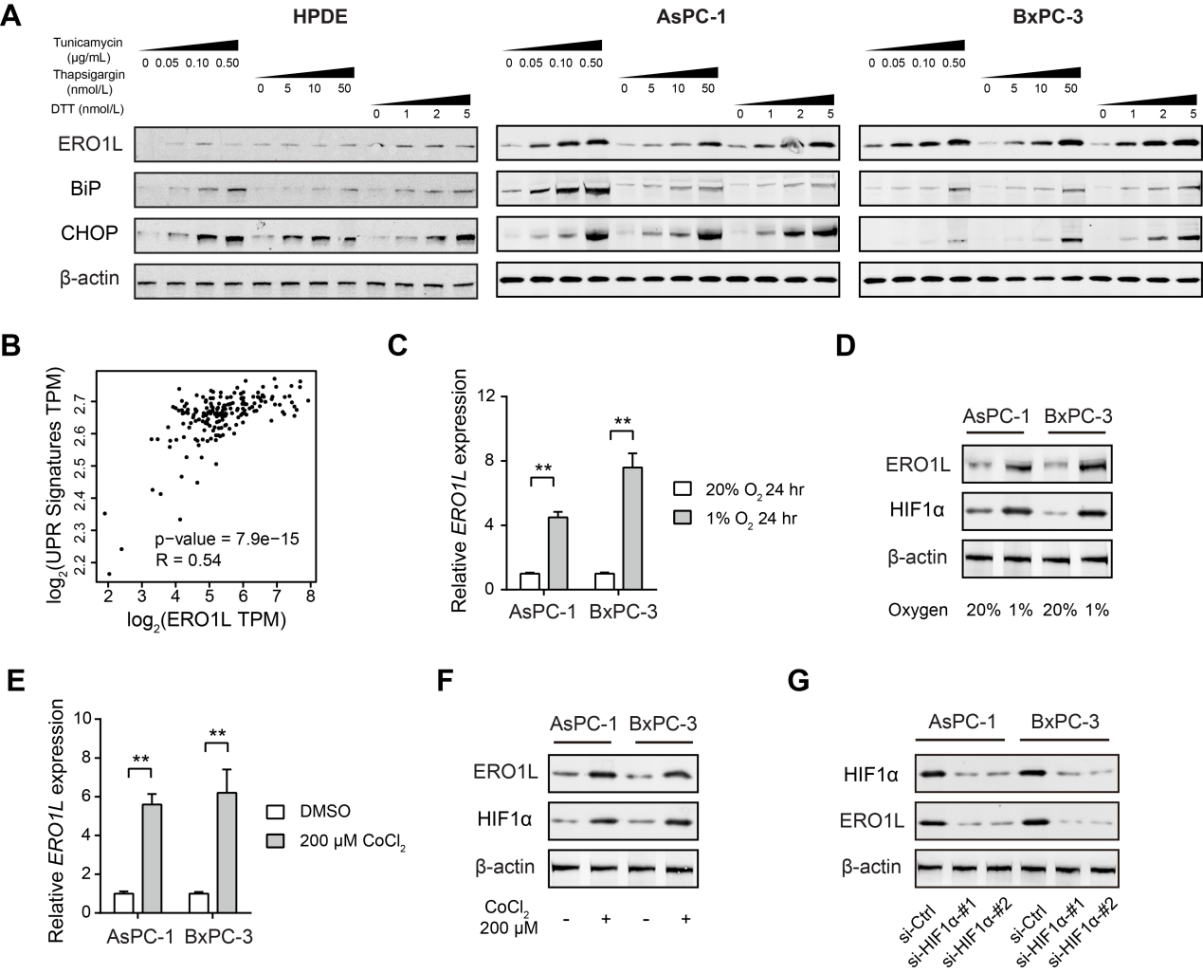
** ERO1L is induced by ER stress in pancreatic cancer.** (**A**) Western blotting analysis of ERO1L protein level after treatment with vehicle (DMSO) or increasing concentrations of three chemical inducers of ER stress (Tunicamycin, Thapsigargin, and DTT) in HPDE, AsPC1 and BxPC3 cells. (**B**) Correlation analyses of expression of ERO1L and unfolded protein response (UPR) gene expression signature in TCGA cohort. (**C**) Detection of ERO1L mRNA level in AsPC1 and BxPC3 cells under hypoxia (1% O_2_) and normoxia (20% O_2_) condition (n = 3). (**D**) Western blotting analysis of ERO1L protein in AsPC1 and BxPC3 cells under hypoxia (1% O_2_) and normoxia (20% O_2_) condition. (**E**) Real-time qPCR analysis of ERO1L expression in AsPC1 and BxPC3 cells after exposure to 100 µM CoCl_2_ for 24 h. (**F**) Western blotting analysis of ERO1L protein in AsPC1 and BxPC3 cells after exposure to 100 µM CoCl_2_ for 24 h. (**G**) Comparison of ERO1L expression in si-Ctrl and si-HIF1α AsPC1 and BxPC3 cells under hypoxia (1% O_2_) condition. **P < 0.01.

It has been reported that ERO1L is transcriptionally regulated by the ER unfolded protein response 25, 26. To distinguish this possibility in PDAC, we assessed whether ERO1L expression is induced upon treatment with three established chemical inducers of ER stress: thapsigargin, tunicamycin, and dithiothreitol (DTT). Two cell lines (AsPC-1 and BxPC-3) with lower endogenous expression of ERO1L were subjected for analysis (Figure S1A). As shown in Figure 3A, ERO1L protein levels in AsPC-1 and BxPC-3 cells were markedly increased by all three ER stressors in a dose-dependent manner. As the next line of evidence, ERO1L and ER stress-related genes were strongly correlated in their expression (Figure 3B). Thirdly, we pharmacologically inhibited PERK-EIF2α with GSK2656157 in AsPC-1 and BxPC-3. The result showed that GSK2656157 (1 μmol/L) largely abrogated ERO1L expression in response to Tunicamycin-induced ER stress (Figure S1B).

One of the key factors that contribute to the poor clinical outcomes of PDAC is the extent of hypoxia within the tumor tissue. Hypoxia is also known to induce ER stress due to the accumulation of misfolded proteins, which activate the unfolded protein response (UPR) 27. Therefore, we wondered whether ERO1L is induced by hypoxia in PDAC. To address this issue, AsPC-1 and BxPC-3 cells were cultured under both normoxic and hypoxic conditions for 24 h and subjected to ERO1L expression analysis. The result showed that ERO1L level was upregulated by hypoxia at both the mRNA and protein levels (Figure 3C-D). Consistently, a chemical inducer of HIF1α, CoCl_2_, phenocopied the effect induced by hypoxia (Figure 3E-F). Furthermore, siRNA-mediated knockdown of HIF1α significantly attenuated ERO1L expression in AsPC-1 and BxPC-3 cells under hypoxic conditions (Figure 3G). Collectively, these data suggest that ERO1L might be induced by ER stress in PDAC.

### Genetic silencing or pharmacological inhibition of ERO1L suppresses tumor growth in PDAC

**Figure 4:**
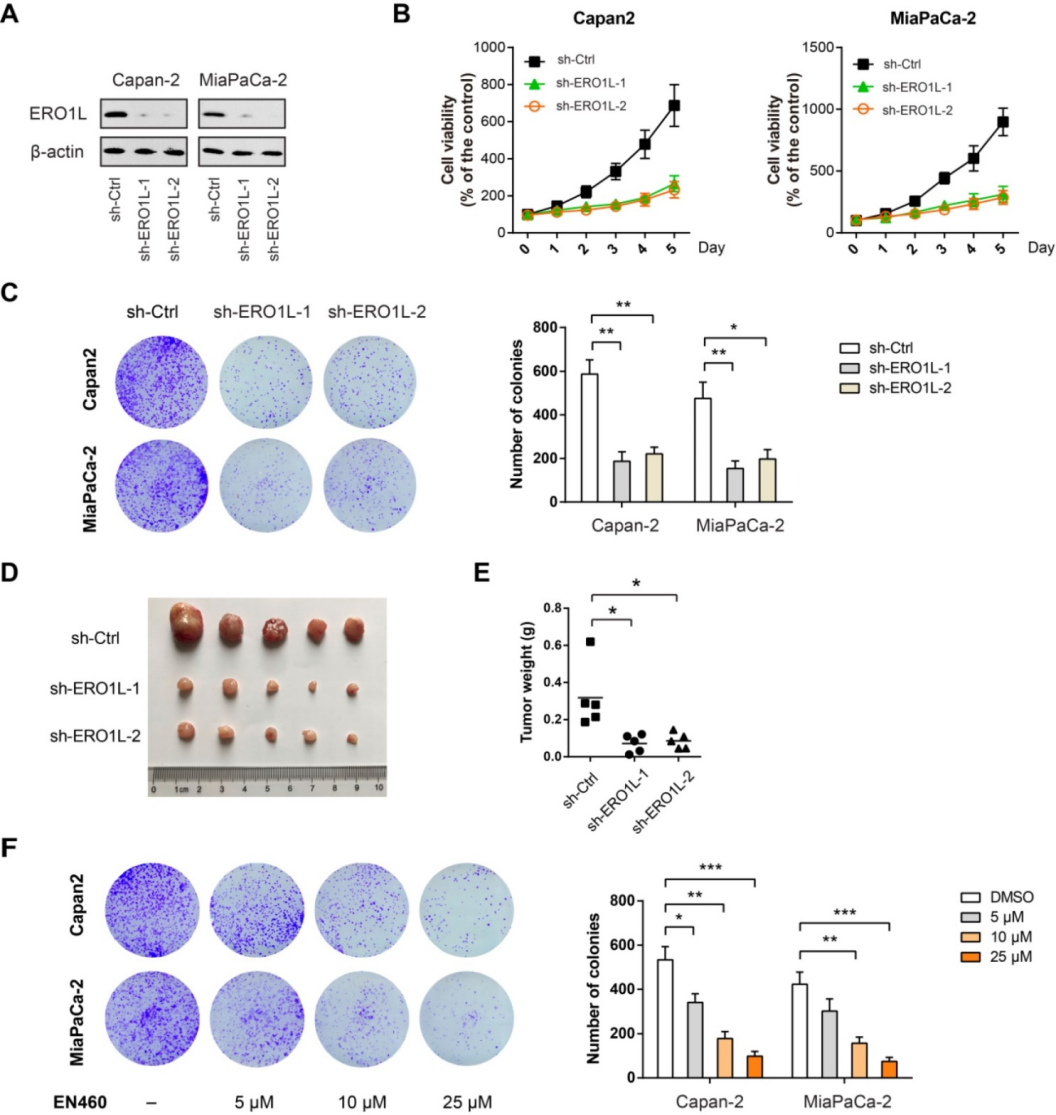
** Genetic silencing or pharmacological inhibition of ERO1L suppresses tumor growth in PDAC.** (**A**) Western blotting analysis of ERO1L knockdown efficiency in Capan-2 and MiaPaCa-2 cells. (**B**) Cell Counting Kit-8 assay analysis of the effect of ERO1L knockdown on Capan-2 and MiaPaCa-2 cell proliferation (n = 3). (**C**) The long-term effect of ERO1L knockdown in Capan-2 and MiaPaCa-2 cells was tested by plate colony formation assay (n = 3). (**D-E**) Capan-2-sh-Ctrl and Capan-2-sh-ERO1L cells were subcutaneously injected into the left and right hind limbs of 5 nude mice; gross xenografts (D) and tumor weights (E) were shown. (**F**) The long-term effect of ERO1L inhibitor EN460 on Capan-2 and MiaPaCa-2 cell proliferation was revealed by colony formation assay (n = 3). *P < 0.05; **P < 0.01; ***P < 0.001.

Next, we studied the role of ERO1L in PDAC progression by loss-of-function studies. Towards this end, we first used a short hairpin RNA (shRNA) strategy to silence ERO1L in two PDAC cell lines (Capan-2 and MiaPaCa-2) with high ERO1L protein level (Figure S1A). Two shRNAs against *ERO1L* led to a > 80% reduction of ERO1L protein expression in both cell lines (Fig. 4A). Cells with ERO1L knockdown exhibited a significant decrease in cell viability compared with their respective non-target controls (Figure 4B). Moreover, ERO1L knockdown exhibited a long-term suppressive effect on cell proliferation as revealed by plate colony formation assay (Figure 4C). In subcutaneous xenograft model, ERO1L knockdown resulted in significantly retarded tumor growth as evidenced by tumor weight (Figure 4D-E). EN460 is a known selective inhibitor of ERO1L that targets its enzymatic activity. Expectedly, pharmacological inhibition of ERO1L by EN460 reduced cell viability and proliferation in a dose-dependent manner (Figure 4F). EN460 is able to inhibit other FAD-containing enzymes including MAO-A, MAO-B, and LSD1, therefore, we could not fully rule out the possible contribution of these targets to EN460-mediated inhibitory effects 28. However, our findings, at least in part, support a role for ERO1L in promoting PDAC proliferation and growth.

### Forced ERO1L expression promotes PDAC growth *in vitro* and *in vivo*

**Figure 5:**
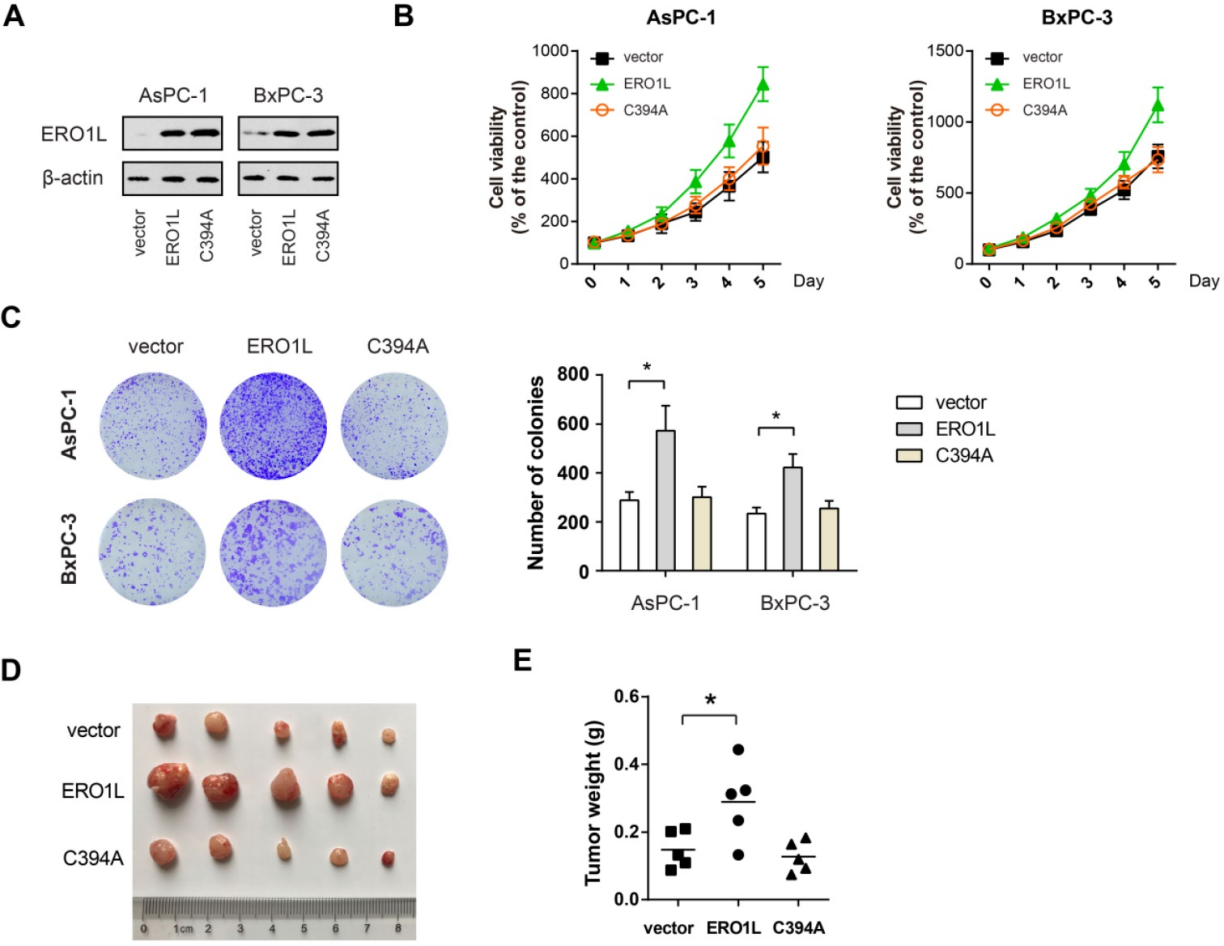
** ERO1L activity is essential for its growth-promoting role in PDAC.** (**A**) Western blotting analysis of AsPC1 and BxPC3 cells after overexpressing either wide type ERO1L or its inactive mutant C394A. (**B**) Cell viability of ERO1L-WT or ERO1L-C394A overexpressing AsPC1 and BxPC3 cells (n = 3). (**C**) The long-term effect of ERO1L-WT or ERO1L-C394A overexpression in AsPC1 and BxPC3 cells was revealed by colony formation assay (n = 3). (**D-E**) AsPC1-vector, AsPC1-ERO1L-WT, and AsPC1-ERO1L-C394A cells were subcutaneously injected into the left and right hind limbs of 5 nude mice; gross xenografts (D) and tumor weights (E) were shown. *P < 0.05.

To further confirm the role of ERO1L in PDAC cell proliferation, we overexpressed ERO1L and its inactive mutant C394A in AsPC-1 and BxPC-3 cells. The overexpression efficiency was verified by Western blotting (Figure 5A). The result showed that ectopic ERO1L expression remarkably promoted the cell viability and colony formation capacity of AsPC-1 and BxPC-3 cells (Figure 5B-C). Consistently, the xenograft experiment uncovered that enforced ERO1L expression significantly increased the tumor burden (Figure 5D-E). Interestingly, *in vitro* and *in vivo* growth advantages were not observed by ectopic expression of the non-functional mutant form of ERO1L (Figure 5B-E), suggesting that the effects observed were redox-dependent and not due solely to protein accumulation of ERO1L in the ER.

### ERO1L regulates Warburg effect to facilitate tumor growth

**Figure 6:**
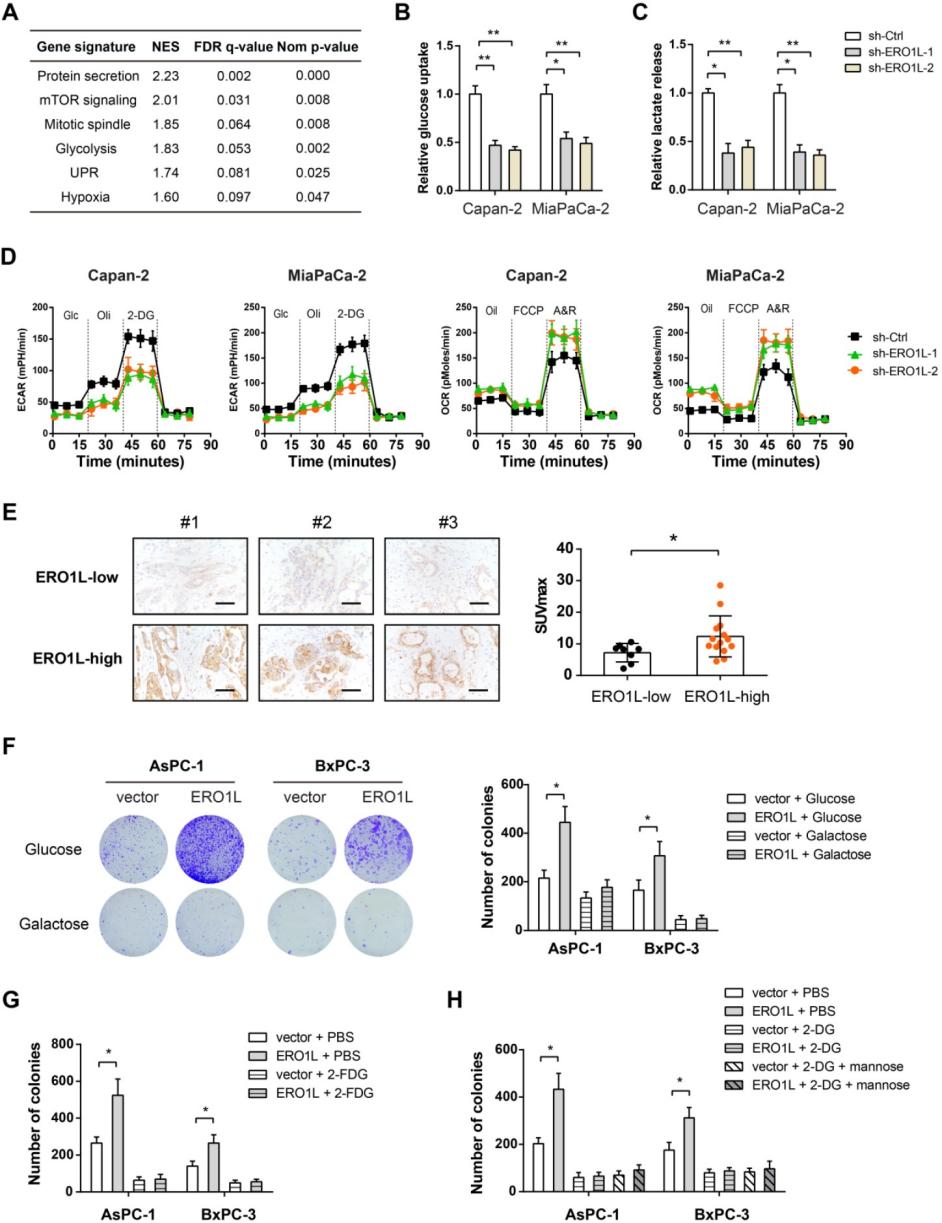
** ERO1L promotes the Warburg effect in pancreatic cancer cells.** (**A**) Gene set enrichment analysis showed significant gene sets related to ERO1L expression; NES, normalized enrichment score; false discovery rate (FDR) was set at 0.25. (**B**) Measurement of glucose uptake in sh-Ctrl and sh-ERO1L Capan-2 and MiaPaCa-2 cells (n = 3). (**C**) Measurement of lactate production in sh-Ctrl and sh-ERO1L Capan-2 and MiaPaCa-2 cells (n = 3). (**D**) Detection of the extracellular acidification rate (ECAR) and oxygen consumption rate (OCR) in sh-Ctrl and sh-ERO1L Capan-2 and MiaPaCa-2 cells (n = 3). (**E**) Representative images of ERO1L expression in tumor tissues from PDAC patients who received preoperative ^18^F-FDG PET/CT examination; scale bar: 50 µm; the difference in the SUVmax value between ERO1L-high and ERO1L-low groups was analyzed. (**F**) AsPC1 and BxPC3 cells were cultured in media containing galactose but no glucose; plate colony formation experiment was performed to determine the anchorage-dependent tumor growth. (G) Effect of 2-FDG on the plate colony formation ability of ov-vector and ov-ERO1L AsPC1 and BxPC3 cells. (**G**) Effect of 2-DG on the plate colony formation ability of ov-vector and ov-ERO1L AsPC1 and BxPC3 cells in the presence or absence of 10 mM mannose. *P < 0.05 and **P < 0.01.

To pursue the mechanism by which ERO1L promotes tumor growth in PDAC, we performed gene set enrichment analysis (GSEA) by using RNA-seq data from TCGA cohort. Based on the median *ERO1L* mRNA expression value, we divided the cohort into ERO1L-high group and ERO1L-low group. Notably, GSEA result showed that ERO1L was closely associated with glycolysis indicative of the possibility of ERO1L in regulating the Warburg effect (Figure 6A). Indeed, ERO1L knockdown led to a dramatic decrease in glucose uptake and lactate release in PDAC cells (Figure 6B-C). By detecting extracellular acidification rate and oxygen consumption rate with Seahorse XF Analyzers, we found that ERO1L contributed to a shift from mitochondrial oxidative phosphorylation to aerobic glycolysis (Figure 6D). An opposite result was found in gain-of-function study (Figure S2). In a cohort of 22 PDAC patients who received preoperative ^18^F-FDG PET/CT imaging, we found that the SUVmax was considerably higher in samples with higher ERO1L expression than in those with lower ERO1L expression (Figure 6E). Moreover, a close correlation was found between ERO1L expression and glycolytic components, including glucose transporter, glycolytic enzymes, and lactate transporter (Figure S3). The Warburg effect can enable rapid cell proliferation by providing abundant cellular buildings for biosynthetic pathways, including nucleotides, lipids, and nonessential amino acids 29-31. Therefore, we investigated whether the effect of ERO1L on PDAC is associated with its ability to modulate the Warburg effect. We grew AsPC-1 and BxPC-3 cells in culture medium containing galactose instead of glucose. The rate at which galactose enters glycolysis is a much lower than that of glucose. Notably, galactose largely compromised ERO1L-mediated upregulation of glycolysis and down-regulation of OXPHOS (Figure S4A). As anticipated, ectopic expression of ERO1L failed to promote cell proliferation under this condition (Figure 6F).

It is well known that 2-deoxy-D-glucose (2-DG) and 2-fluoro-deoxy-D-glucose (2-FDG) are two potent glycolytic inhibitors 32. Indeed, both 2-FDG and 2-DG abrogated the growth-promoting activities of ERO1L in PDAC (Figure 6G-H and Figure S4B-C). Different from 2-FDG, 2-DG at a concentration of 5 mM can induce ER stress by interfering with protein glycosylation 33. Moreover, as previously described 33, addition of mannose reversed the ER stress effect and compromised ERO1L expression induced by 2-DG (Figure S4D). Moreover, addition of mannose reversed the ER stress effect and compromised ERO1L expression induced by 2-DG (Figure S4D). However, mannose failed to reverse 2-DG-mediated glycolysis inhibition (Figure S4E). Collectively, these data indicate that ER stress-induced ERO1L expression promotes glycolysis-dependent tumor growth in PDAC.

### Correlation between UPR and glycolysis genetic signature in human PDAC

**Figure 7:**
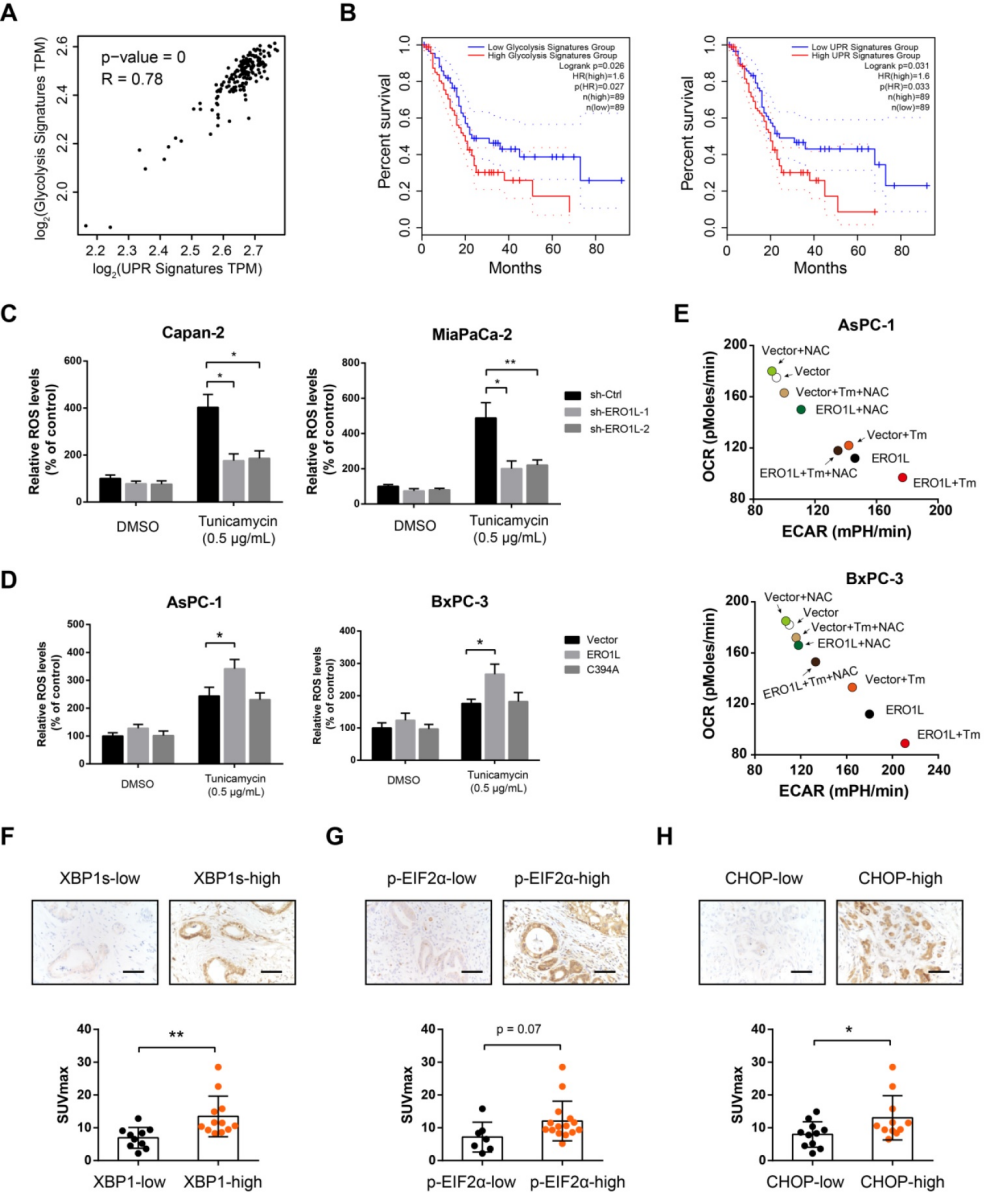
** Correlation between UPR and glycolysis genetic signature in human PDAC.** (**A**) Correlation analyses between UPR and glycolysis gene expression signature in TCGA cohort. (B) Kaplan-Meier graphs showing the association of UPR and glycolysis gene expression signature with PDAC patient survival. (**C**) Effects of ERO1L knockdown on ROS generation under the condition of ER stress in Capan-2 and MiaPaCa-2 cells. (**D**) Effects of ERO1L-WT or ERO1L-C394A overexpression on ROS generation under the condition of ER stress in AsPC-1 and BxPC-3 cells. (**E**) Effect of ERO1L overexpression on PDAC cell glycolysis and OXPHOS in the presence or absence of ER stress and N-acetyl cysteine (0.2 mM) treatment. (F-H) Representative images of spliced XBP1 (**F**), phosphorylated eIF2α (**G**), and CHOP (**H**) expression in tumor tissues from PDAC patients who received preoperative ^18^F-FDG PET/CT examination; scale bar: 50 µm; the difference in the SUVmax value between indicated groups was analyzed. *P < 0.05 and **P < 0.01.

In response to ER stress, ERO1L maintains the activity of oxidative protein folding to improve cell fitness to challenges of high levels of protein misfolding. However, the byproduct of ERO1L, H_2_O_2_, should be safely addressed to avoid cancer cell apoptosis. Glucose flux through aerobic glycolysis and the pentose phosphate pathway (PPP) provides high levels of ATP, NADPH, and ultimately, glutathione (GSH), which is essential for H_2_O_2_ equilibration. Therefore, we hypothesized that UPR and tumor glycolysis is coupled to enable rapid cell proliferation. Indeed, there is a close correlation between UPR and glycolysis gene expression signature (Figure 7A). Specifically, survival analysis using TCGA data set demonstrated that tumors with an elevated UPR or glycolysis signature displayed shorter overall survival than those with a lower UPR or glycolysis signature (Figure 7B). Furthermore, ERO1L knockdown led to a significant decrease in ROS level under ER stress (Figure 7C). In contrast, opposite result was observed in gain-of-function study (Figure 7D). Neutralizing of ROS level by N-acetyl cysteine largely compromised the effects of ERO1L in promoting aerobic glycolysis (Figure 7E) and tumor growth (Figure S5A). Treatment with GSH inhibitors Buthionine-[S, R]-sulfoximine (BSO) lead to a significant increase in ROS levels and a transient increase in glycolytic capacity (Figure S5B-C). However, BSO treatment did not boost ROS-mediated long-term promotion of cell proliferation upon ERO1L overexpression, suggesting that high ROS levels induced by BSO may exert an oxidative stress on the PDAC cell that can ultimately decrease cell viability. To more directly assess our hypothesis, we determined whether glycolysis status is related to UPR signaling pathways known to be activated by ER stress. In clinical samples, we found that spliced XBP1, phosphorylated eIF2α, and CHOP expression were significantly upregulated in tumors with high glycolytic capacity as indicated by SUVmax (Figure 7F-H). Taken together, these data suggest that increased glycolysis might be a characteristic of PDAC cells that have undergone modest ER stress.

## Discussion

In this study, we comprehensively characterized the expression profile and prognostic value of ERO1L in human cancers, especially PDAC. First, we revealed that UPR-dependent ERO1L expression acts as an independent prognostic factor for PDAC patients. Using a combination of gain- and loss-of-function strategies, we showed that ERO1L confers robust growth-promoting effects. Finally, we demonstrated that ERO1L-induced growth advantage is largely dependent on enhanced aerobic glycolysis. Taken together, these findings uncover a mechanistic axis directly linking the UPR, ERO1L, aerobic glycolysis, and associated tumor growth in PDAC.

Cancer cells are exposed to diverse intrinsic and extrinsic factors that disrupt protein homeostasis, producing ER stress. To counter these situations, cancer cells employ the dynamic intracellular UPR signaling pathway to restore the ER homeostasis 34. ERO1L provides an efficient oxidative protein folding process and therefore is a determinant for ER homeostasis. In this study, we revealed that ERO1L expression is significantly elevated by activation of UPR. Consistent with two previous reports 35, 36, we also found that ERO1L can be induced by hypoxia, which is known to induce ER stress. The UPR-dependent elevation of ERO1L might be a reflection of the global enhancement of the entire secretory apparatus in response to ER stress. Since PDAC is characterized by intense hypoxia and high UPR activation status, we therefore assessed ERO1L expression in a preclinical PDAC mouse model and human PDAC. These analyses together with previous findings confirmed that ERO1L is highly expressed in PDAC and associated with a poor clinical outcome in patients with PDAC.

ERO1L plays versatile oncogenic functions in human cancers, including rapid cell proliferation, increased cell invasion, promotion of angiogenesis, and immune escape 16, 36, 37. Using the CRISPR/Cas9 approach, Gupta et al. showed that ERO1L KO significantly suppresses PDAC cell proliferation and colony-forming potential 35. Similarly, we found that shRNA-mediated ERO1L knockdown inhibits cell viability and colony-forming ability of two PDAC cells. Interestingly, we for the first time reported that ERO1L plays a glycolysis-dependent growth-promoting role in PDAC. Blocking glycolysis with 2-DG, 2-FDG or galactose largely abrogated ERO1L-mediated growth-promoting effect. Since 2-DG induces ER stress it might be expected to increase ERO1L expression and thereby make PDAC cells grow even better. However, our results showed that 2-DG lowers colony counts similarly in ERO1L-overexpressing or vector PDAC cells indicates that the glycolytic inhibitory effect of 2-DG at the concentration used (5 mM) overrides any additional increase of growth that may be occurring due to 2-DG-induced ER stress. Subsequently, we noticed a close link between UPR and aerobic glycolysis in PDAC. However, how ERO1L bridges these two cellular processes? First, the byproduct of ERO1L, reactive oxygen species (ROS), is able to regulate the activity of HIF1α, which can directly promote aerobic glycolysis by transcriptional regulation of glycolytic enzymes or glucose transporter 38, 39. Therefore, a positive ERO1L/HIF1α feedback loop may couple UPR to aerobic glycolysis in PDAC. Second, the UPR acts as a double edged-sword; the UPR initially compensates for damage and reestablishes ER homeostasis, but triggers growth inhibition or cell death if UPR outputs are maladaptive or prolonged 6, 7. Thus, increased ROS levels generated by ERO1L must be resolved by reduced glutathione (GSH) to avoid cell apoptosis. Cancer cells maintain pools of reduced GSH using NADPH in part produced by aerobic glycolysis 40. In this scenario, increased aerobic glycolysis might be an intrinsic adaption in response to UPR to boost cell fitness. Moreover, many other pathways can be activated by ERO1L, such as the PI3K-Akt/mTOR, S1PR1/STAT3, and Wnt/β-catenin signaling pathways 16, 19, which are known to promote rapid cell proliferation and Warburg effect. Collectively, more works are warranted to elucidate the influence of ERO1L on pancreatic cancer progression and cellular metabolism.

In conclusion, we have uncovered the expression pattern and prognostic value of ERO1L in PDAC, and have identified a novel function of ERO1L in regulating aerobic glycolysis. Importantly, ERO1L is tightly regulated by UPR, indicating that targeting the UPR-ERO1L-glycolysis axis in combination with standard chemotherapy may improve the effectiveness of anti-PDAC therapies.

## Supplementary Material

Supplementary figures and tables.
