# Supplementary material for: Endoplasmic Reticulum stress-dependent expression of ERO1L promotes aerobic glycolysis in Pancreatic Cancer
Source: Theranostics. 2020 Jul 9;10(18):8400–14. doi: 10.7150/thno.45124 (PMC7381747; doi:10.7150/thno.45124)
Supplement: Supplementary file 1 — Supplementary figures and tables. [file thnov10p8400s1.pdf]

Supplementary information

Table S1: Correlations between ERO1L expression and clinicopathologic parameters in PDAC patients

Clinicopathological parameter	Total 205	Expression of ERO1L		*P*-value
		Low (n=102, %)	High (n=103, %)	
**Age (years)**				
< 65	97	51 (52.6)	46 (47.4)	0.486
≥ 65	108	51 (47.2)	57 (52.8)	
**Gender**				
Male	117	63 (53.8)	54 (46.2)	0.205
Female	88	39 (44.3)	49 (55.7)	
**Tumor location**				
Head	139	70 (50.4)	69 (49.6)	0.881
Body/tail	66	32 (48.5)	34 (51.5)	
**TNM (AJCC)**				
Stage I	38	24 (63.2)	14 (36.8)	0.316
Stage II	132	62 (47.0)	70 (53.0)	
Stage III	21	9 (42.9)	12 (57.1)	
Stage IV	14	7 (50.0)	7 (50.0)	
**Tumor size**				
≤ 3 cm	69	45 (65.2)	24 (34.8)	**0.002**
> 3 cm	136	57 (41.9)	79 (58.1)	
**T classification**				
T1, 2	42	26 (61.9)	16 (38.1)	0.086
T3, 4	163	76 (46.6)	87 (53.4)	
**Lymph node metastasis**				
Absent	136	71 (52.2)	65 (47.8)	0.376
Present	69	31 (44.9)	38 (55.1)	
**Distant metastasis**				
Absent	191	95 (59.7)	96 (50.3)	1.000
Present	14	7 (50.0)	7 (50.0)	
**Vascular invasion**				
Absent	178	92 (51.7)	86 (48.3)	0.215
Present	27	10 (37.0)	17 (63.0)	
**Histological differentiation**				
Well	11	9 (81.8)	2 (18.2)	**0.033**
Moderate/poor	194	93 (47.9)	101 (52.1)	

The bold number represents the *P*-values with significant differences. *P* value was calculated by χ^2 test or Fisher's exact test.

Table S2: Univariate analysis of prognostic parameters for survival in PDAC patients

**Clinical parameters**	**HR**	**95% CI**	***P*-value**
Expression of ERO1L (high vs. low)	1.606	1.142-2.260	**0.004**
Age (≥ 65 years vs. < 65 years)	1.521	1.077-2.149	**0.017**
Gender (male vs. female)	0.734	0.514-1.047	0.088
Tumor location (head vs. body/tail)	1.019	0.708-1.466	0.920
TNM stage (III-IV vs. I-II)	1.267	1.011-1.588	**0.040**
Tumor size (> 3 cm vs. ≤ 3 cm)	2.141	1.182-3.879	**0.012**
T classification (T3, 4 vs. T1, 2)	1.347	0.869-2.086	0.182
Lymph node metastasis (present vs. absent)	1.487	1.049-2.109	**0.026**
Distant metastasis (present vs. absent)	1.945	1.041-3.634	**0.037**
Vascular invasion (present vs. absent)	1.579	0.969-2.572	0.067
Histological differentiation (moderate/poor vs. well)	2.475	1.011-6.058	**0.047**

HR: Hazard ratio; CI: Confidence interval. The bold number represents the *P* value with significant differences.

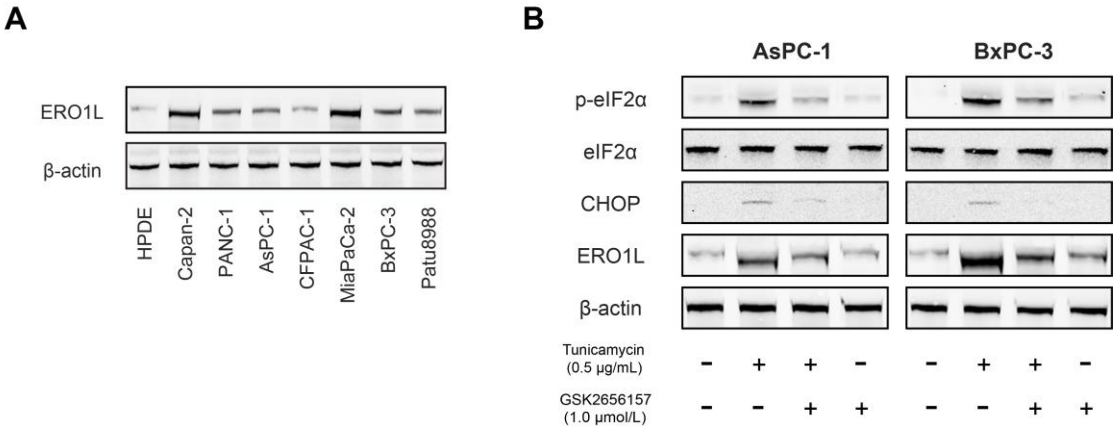

Figure S1. UPR-dependent expression of ERO1L in PDAC cells. (A) Western blotting analysis of ERO1L protein level in seven PDAC cell lines and the non-malignant HPDE cell line; β-actin was used as an internal control. (B) Western blotting analysis of the effect of PERK-EIF2α inhibitor (GSK2656157) on ERO1L protein level in response to Tunicamycin-induced ER stress.

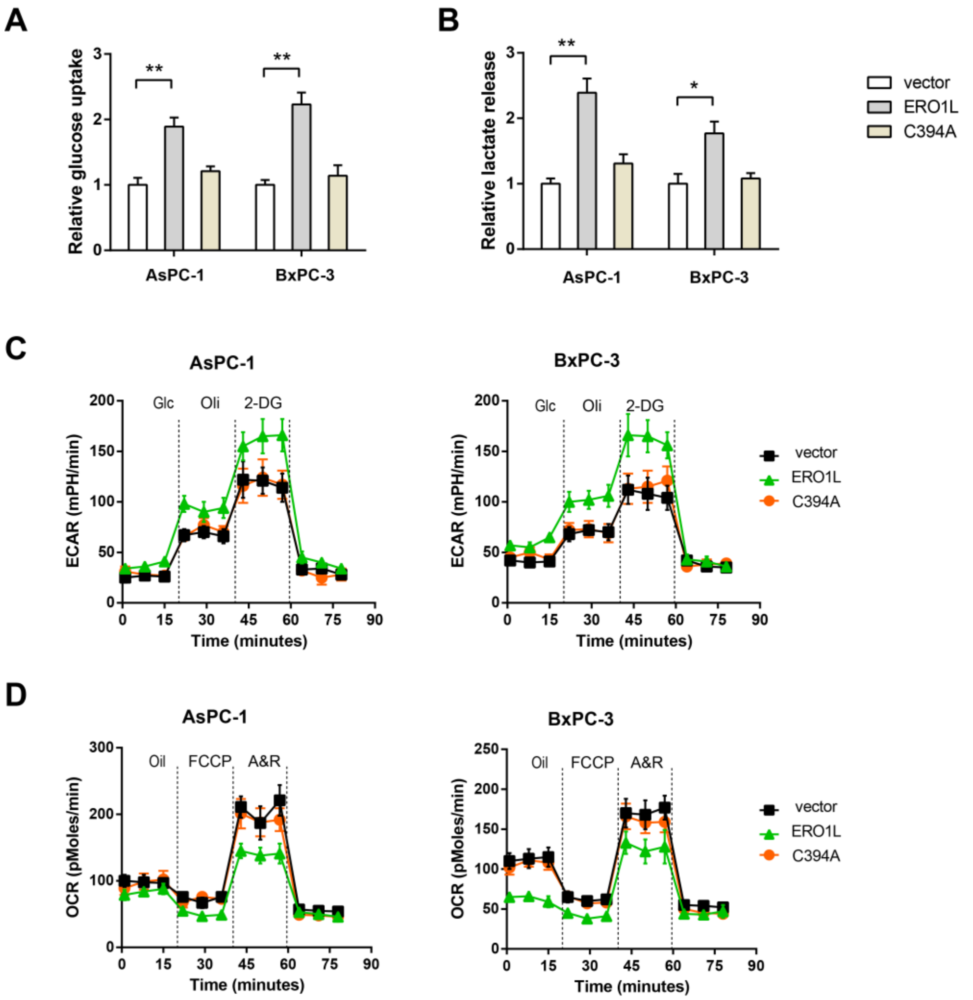

Figure S2. Overexpression of ERO1L promotes the Warburg effect in pancreatic cancer cells. (A) Measurement of glucose uptake in vector, ERO1L-overexpressing and ERO1L-C394A-overexpressing AsPC-1 and BxPC-3 cells (n = 3). (B) Measurement of lactate production in vector, ERO1L-overexpressing and ERO1L-C394A-overexpressing AsPC-1 and BxPC-3 cells (n = 3). (C-D) Detection of the extracellular acidification rate (ECAR, C) and oxygen consumption rate (OCR, D) in vector, ERO1L-overexpressing and ERO1L-C394A-overexpressing AsPC-1 and BxPC-3 cells (n = 3). *P < 0.05 and **P < 0.01.

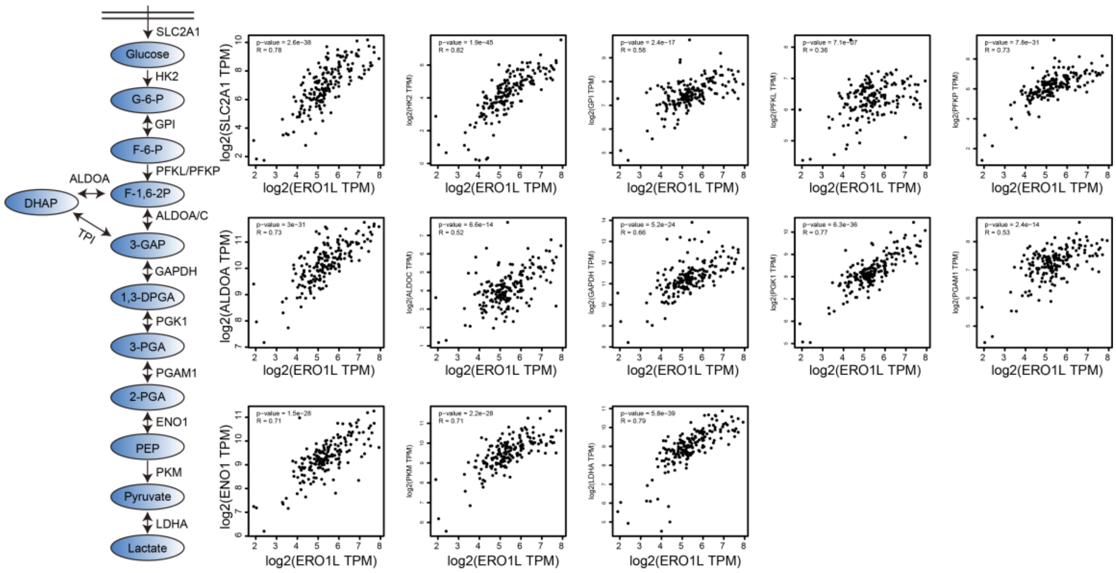

Figure S3. Correlation analysis between ERO1L level and expression of glycolytic components (glucose transporter and glycolytic enzymes) in PDAC samples. Data were derived from TCGA cohort (n = 179).

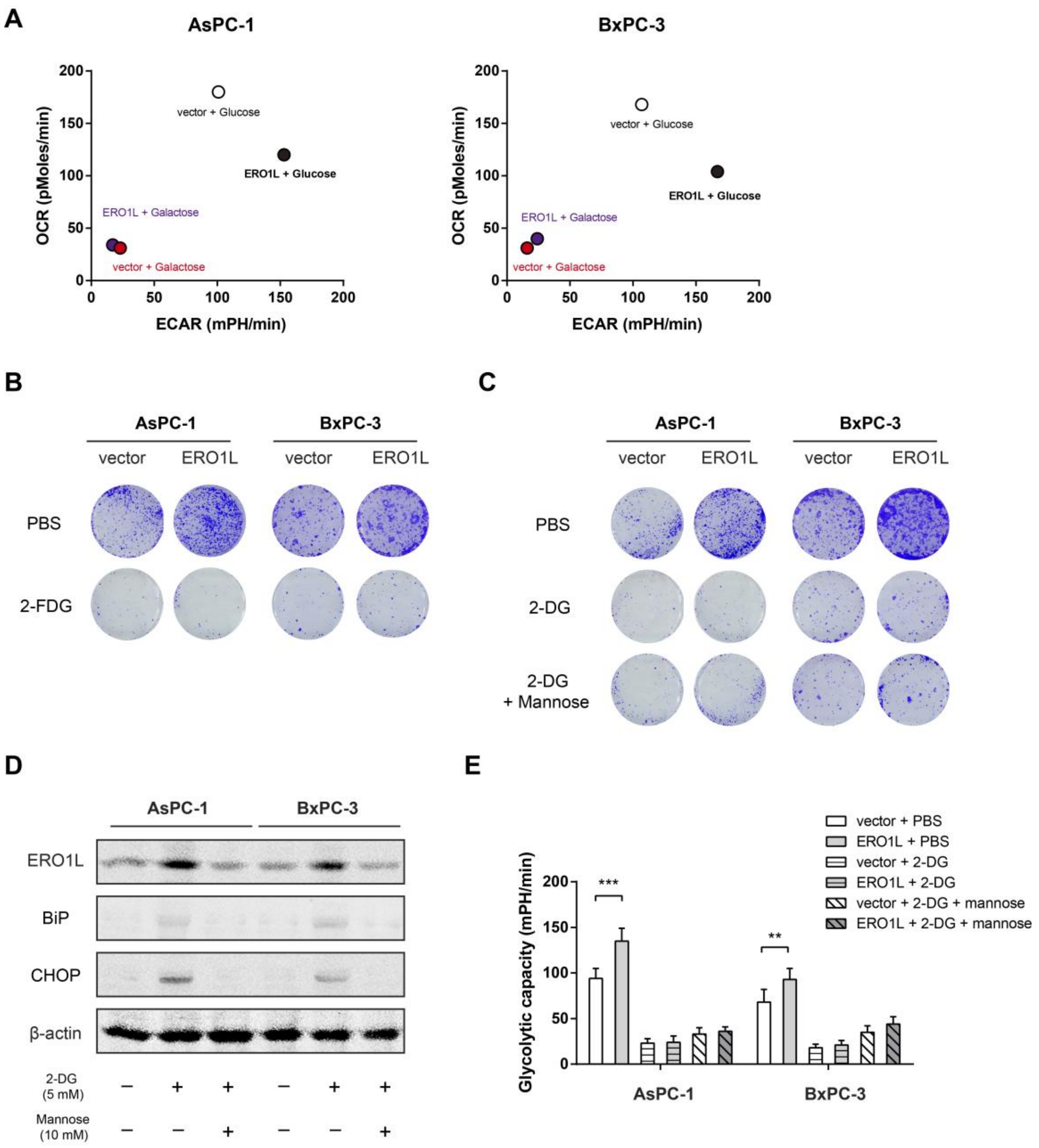

Figure S4. Glycolysis-dependent growth-promoting effect of ERO1L in PDAC. (A) Seahorse analysis of OXPHOS and glycolysis in the presence of galactose by measuring OCR and ECAR in AsPC-1 and BxPC-3 cells (n = 3). (B) Effect of 2-FDG on the plate colony formation ability of ov-vector and ov-ERO1L AsPC1 and BxPC3 cells (n = 3). (C) Effect of 2-DG on the plate colony formation ability of ov-vector and ov-ERO1L AsPC1 and BxPC3 cells in the presence or absence of 10 mM mannose (n = 3). (D) Effect of 2-DG (5 mM) on ER stress and ERO1L expression in the presence or absence of mannose (10 mM). (E) Seahorse analysis of the effect of 2-DG on the glycolytic capacity of AsPC1 and BxPC3 cells in the presence or absence of mannose (n = 9). $^{**}P < 0.01$ and $^{***}P < 0.001$.

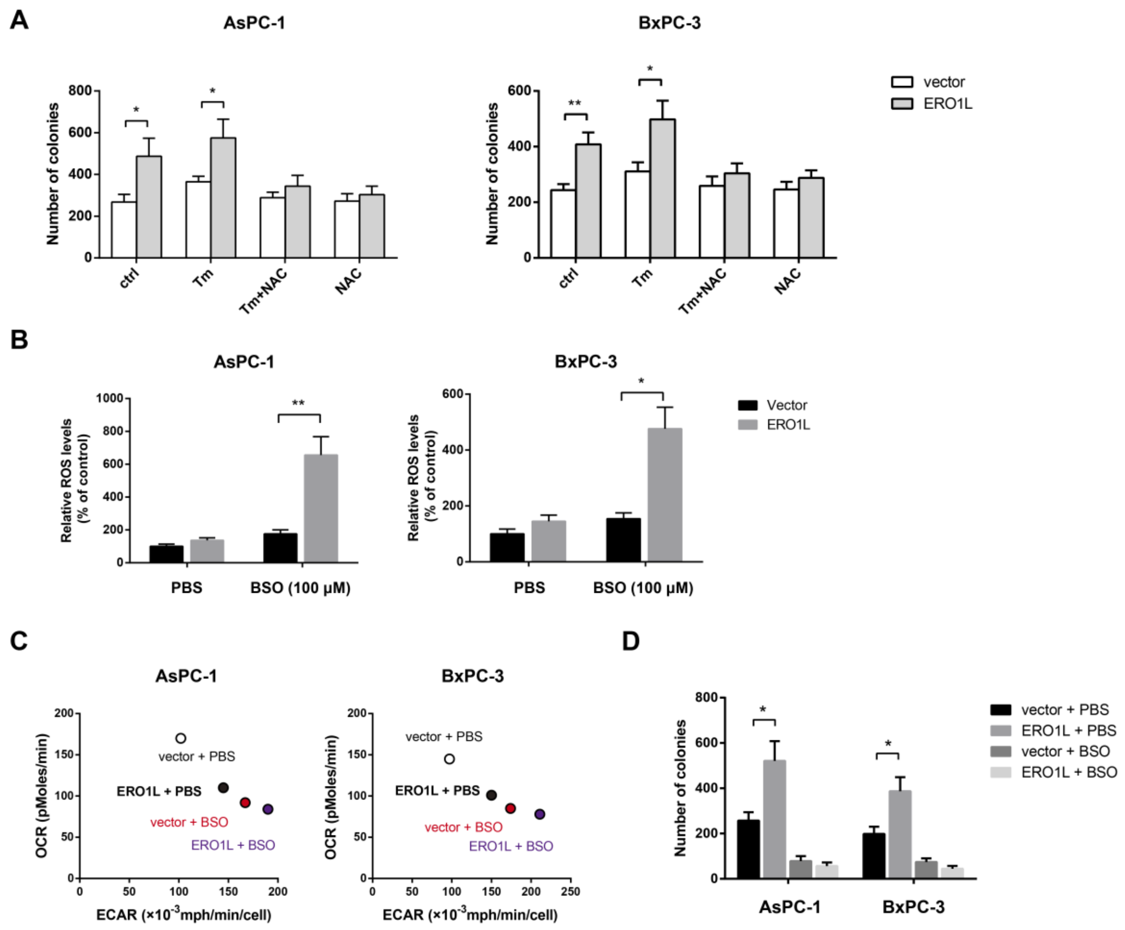

Figure S5. ERO1L oxidoreductase activity is essential for its growth-promoting effect. (A) Neutralizing of ROS levels by N-acetyl cysteine (NAC, 0.2 mM) largely compromised the effect of ERO1L in facilitating tumor growth (n = 3). (B) Effects of GSH inhibitor BSO (100 μM) on ROS generation in ov-vector and ov-ERO1L AsPC-1 and BxPC-3 cells (n = 3). (C) Seahorse analysis of OXPHOS and glycolysis in the presence of BSO treatment (100 μM) by measuring OCR and ECAR in AsPC-1 and BxPC-3 cells (n = 3). (D) Effect of BSO treatment (100 μM) on the plate colony formation ability of ov-vector and ov-ERO1L AsPC-1 and BxPC-3 cells (n = 3). *P < 0.05 and **P < 0.01.
